# Œdema and Anasarca

**Published:** 1916-10-21

**Authors:** 


### ŒDEMA AND ANASARCA.

### Some Overlooked Factors in Causation.

Dropsy of the subcutaneous tissues is one of the evidences of disease which has, from the practitioner's point of view, many satisfactory features. In the first place it is easily recognised. Secondly it is, without question, abnormal. And yet again, in the great majority of cases it is capable of a confident clinical interpretation. Whatever pathological discussions may exist relative to its immediate cause its meaning in practical medicine usually causes little difficulty. The features of the two contrasted types—cardiac dropsy and renal dropsy—are well known and need no discussion. Failure in one of these two directions is the expected interpretation when œdema or anasarca is a prominent feature of the patient's symptomatology. With a localised and especially with a unilateral distribution, the possibility of some local influence producing obstruction to the venous return must of course be considered, as, for example, pressure within the pelvis or abdomen, causing œdema of the lower limbs, or an intrathoracic tumour or aneurism leading to dropsy over the chest wall or in one or other of the upper limbs. But, excluding these more or less obvious conditions, it is to cardiac or to renal disturbance that the diagnosis is inclined when œdema is one of the leading features of the case. So generally true is this remark that, given the existence of œdema, and given also the absence of all other symptoms which throw doubt upon the functional value of the heart or kidneys, the existence of the dropsy is apt to create some degree of bewilderment. The suggestion arising from experience is that dropsy means either cardiac or renal disease. When in an individual case the evidence negatives such an interpretation there arises inevitably some sense of diagnostic difficulty.

### The Rarer Causes of Œdema.

There are, however, clinical conditions other than heart disease and kidney disease of which subcutaneous œdema is at times a prominent feature; and when the diagnostic position which has just been presented exists it is well to keep these less common causes of dropsy in view. This is all the more necessary because the dropsy may make such a prominent impression on the patient's mind that he puts this symptom in the foreground of his complaint, and he may indeed deny the existence of all other symptoms. It is therefore only by careful clinical investigation that facts are discovered which lead to a resolution of the mystery.

Prominent among the less common causes of subcutaneous œdema is a depreciated condition of the quality of the blood. Even simple œdema of the chlorotic type may be attended with puffiness about the ankles, and sometimes the dropsy is much more prominent. Again, malignant disease may be responsible for a similar result, not by local pressure and venous obstruction but by the alteration it produces in the blood quality. And if, as is sometimes the case, the malignant growth does not come within reach of physical examination, the dropsy may appear the chief feature of the case, and may thus present a truly puzzling quality. But while simple anæmia and malignant disease have their place as possible causes of œdema, it is the definite blood diseases which are apt to lead to dropsies in a highly pronounced form. Pernicious anæmia and leucocythæmia are conspicuous here; and in most instances, if these possibilities are borne in mind, the diagnosis is easily resolved. A careful examination of the blood shows facts which are not open to any doubtful interpretation, and the patient who regarded dropsy as the one ground of complaint is found to be the subject of a very definite and serious disease. Failing disease of the heart or of the kidneys as an explanation of a given case of dropsy, the possibility of a disease of the blood should be recalled and the investigation necessary to confirm or to contradict this should be undertaken.

### Peripheral Neuritis.

Still another group of cases in which œdema may take a prominent place is found among nervous diseases. The reference is to cases of peripheral neuritis, and more particularly, it would appear, to the alcoholic variety of peripheral neuritis. Here, again, it must be asserted that the dropsy, in the patient's view, may be the principal or even the sole cause for complaint. Hence it will only be by thorough examination that the true meaning will be discovered. In short, peripheral neuritis must be added to disorders of the blood as one of the possible causes of an œdema which exists in spite of the absence of all evidence of disease of the heart or disease of the kidneys. This broad proposition needs to be lodged in the memory, and did it receive due recognition there would be fewer cases of œdema for which no sufficient cause can be discovered. Other still more exceptional causes of œdema no doubt exist, but they appear in operation so rarely that they hardly affect the practical position.

### A Therapeutic Hint.

Two observations bearing on treatment may be added. The first is that when œdema occurs in a case of granular kidney its explanation is not the renal lesion but the cardiac failure which is a consequence of this lesion. Hence the treatment of such a case is the treatment not of renal disease but of cardiac failure. A second remark relates to the use of Southey's tubes. No doubt a widespread œdema may often be removed under rest in bed and the use of diuretics. But not infrequently this discipline is pursued in vain. The wise course is to give it a fair chance but not unduly to prolong it. In an obstinate condition it is far better to take the bold course and to drain the tissues mechanically. However the result is to be explained, it will often be found that after removal of the fluid the kidneys will take on free action though previously the classical remedies have been used in vain.

